# A Comparison of Clinical Manifestations and Prognoses Between Patients with Hepatocellular Carcinoma and Child–Pugh Scores of 5 or 6

**DOI:** 10.1097/MD.0000000000000348

**Published:** 2014-12-02

**Authors:** Hung-Hsu Hung, Yee Chao, Yi-You Chiou, Chung-Pin Li, Rheun-Chuan Lee, Teh-Ia Huo, Yi-Hsiang Huang, Gar-Yang Chau, Chien-Wei Su, Yi-Chen Yeh, Han-Chieh Lin, Shou-Dong Lee, Jaw-Ching Wu

**Affiliations:** From the Division of Gastroenterology (H-HH, S-DL), Department of Medicine, Cheng Hsin General Hospital; Faculty of Medicine, School of Medicine (H-HH, YC, Y-YC, C-PL, R-CL, G-YC, C-WS, Y-CY, H-CL, S-DL); Institute of Clinical Medicine and Genomic Research Center (H-HH, Y-HH, J-CW), National Yang-Ming University; Division of Chemoradiotherapy (YC), Department of Oncology Medicine; Division of Gastrointestinal Radiology (Y-YC), Department of Radiology; Division of Gastroenterology (C-PL, T-IH, Y-HH, C-WS, H-CL), Department of Medicine; Division of Pediatric Radiology (R-CL), Department of Radiology, Taipei Veterans General Hospital; Institute of Pharmacology (T-IH), School of Medicine, National Yang-Ming University; Division of General Surgery (G-YC), Department of Surgery; Department of Pathology and Laboratory Medicine (Y-CY); and Division of Translational Research, Department of Medical Research, Taipei Veterans General Hospital, Taipei, Taiwan (J-CW).

## Abstract

The objective of this work is to compare the outcomes between the Child–Pugh score 5 (A5 group) and Child–Pugh score 6 (A6 group) in patients with hepatocellular carcinoma (HCC).

Whether HCC patients with A5 and A6 groups have different prognoses is still obscure.

We enrolled 2462 consecutive treatment-naive HCC patients from 2007 to 2012. Among them, 1486 patients had Child–Pugh grade A, including 1016 in the A5 group and 470 in the A6 group. Factors in the prognoses were analyzed by multivariate analysis.

Compared with those in the A6 group, patients in the A5 group were younger, had higher proportions of tumors within the Milan criteria, and more of them underwent curative therapies. The cumulative survival rates at 5 years were 51.3% and 37.1% for patients in the A5 and A6 groups, respectively (*P* < 0.001). Multivariate analysis showed that the independent risk factors associated with poor overall survival were nonhepatitis C virus carrier, serum albumin ≤4 g/dL, aspartate aminotransferase >45 U/L, α-fetoprotein >20 ng/mL, multinodularity, tumor size >3 cm, vascular invasion, and noncurative therapies, but not the Child–Pugh numeric score. The Child–Pugh numeric score had a significant prognostic effect only in patients who had tumors beyond the Milan criteria and received noncurative therapies.

HCC patients with A5 group had a better overall survival rate than those with A6 group due to the early tumor stage and higher rate of receiving curative treatments. Tumor factors and treatment modalities were more important than the Child–Pugh numeric score.

## INTRODUCTION

Hepatocellular carcinoma (HCC) is one of the leading causes of cancer mortality in the world.^1,2^ In recent decades, the outcomes of patients with HCC have been improving, but they are still not satisfactory.^3–7^ Factors affecting the prognoses of HCC include patient factors (such as age, sex, and performance status), tumor factors (tumor size, number of tumors, vascular invasion, tumor cell differentiation, etc.), and liver functional reserve (eg, Child–Pugh score, portal hypertension, and platelet count).^8–13^

To date, more than 10 staging systems have been proposed for HCC.^14,15^ Among them, the Barcelona Clinic Liver Cancer (BCLC) staging system is recommended as a treatment allocation guideline because of its excellent prognostic stratification.^16^ The treatment options in HCC patients are dependent on not only the tumor stages, but also the liver functional reserve.^17^ Child–Pugh scores are widely applied to estimate liver functional reserve in many HCC staging systems as part of the predictors. The survival rates are different in each Child–Pugh grade and associated with different treatment modalities.^3^ Consequently, the Child–Pugh grade has been enrolled in the BCLC staging system for the selection of treatment modalities.^16^

Several studies have been conducted to assess the prognoses and efficacy of treatment modalities in HCC patients with Child–Pugh grades B or C.^18–20^ They found that the Child–Pugh numeric score determined patients’ outcomes.^18^ Moreover, for patients at an intermediate tumor stage, locoregional treatment could provide good survival in Child–Pugh grade B patients, especially for those with a Child–Pugh score of 7.^18^ Even in patients with Child–Pugh grade C, the survival rates were significantly higher in patients in the treated group than those in the untreated group.^19,21^ This suggested that prognosis could still be improved by nontransplant treatments in patients with higher Child–Pugh scores.

Patients with well-preserved liver function and Child–Pugh grade A are considered suitable for all treatment modalities. It is recommended that they mainly be treated according to tumor stage with regard to factors such as tumor size, the number of tumors, vascular invasion, and extrahepatic metastasis.^18^ However, there are limited data regarding Child–Pugh grade A patients stratified by Child–Pugh score 5 (A5 group) and Child–Pugh score 6 (A6 group). This study strived to compare the clinical manifestations, treatment modalities, and outcomes between A5 and A6 HCC patients.

## MATERIALS AND METHODS

### Patients and Follow-Up

**FIGURE 1:**
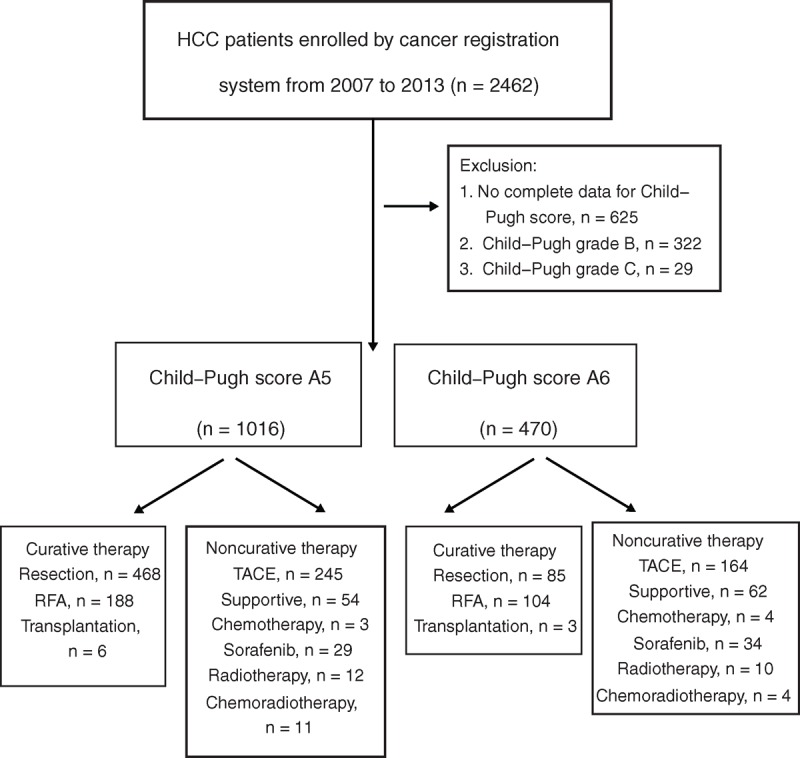
The study flow chart. HCC = hepatocellular carcinoma, RFA = radiofrequency ablation therapy, TACE = transarterial chemoembolization.

This prospectively conducted, retrospectively analyzed cohort study enrolled 2462 consecutive treatment-naive patients who fulfilled the diagnostic criteria of HCC by the American Association for the Study of Liver Diseases (AASLD consensus, 2005) and who were enrolled in the cancer registration system at Taipei Veterans General Hospital from November 2007 to February 2013 (Figure 1).^22^ All of the patients were followed-up every 3 months until their last visit in our hospital, death, or August 31, 2013. All patients underwent clinical, laboratory, and ultrasound assessment to establish the liver cirrhosis severity by using the modified Child–Pugh score (Table 1).^23^ After excluding patients without complete data for Child–Pugh scores (625 patients) and those with Child–Pugh grade B (322 patients) and Child–Pugh grade C (29 patients), a total of 1486 Child–Pugh grade A patients were enrolled for the final analysis. The etiologies of HCC were as follows: chronic hepatitis B virus (HBV) infection (n = 747, 50.3%), chronic hepatitis C virus (HCV) infection (n = 370, 24.9%), dual HBV and HCV infections (n = 48, 3.2%), alcoholism (n = 42, 2.8%), nonalcoholic steatohepatitis (NASH) (n = 184, 12.4%), and cryptogenic cirrhosis (n = 95, 6.4%), respectively. Among them, 1016 patients had A5 group, and the remaining 470 patients had A6 group. These patients were further classified according to curative and noncurative treatment modalities.

**TABLE 1:**
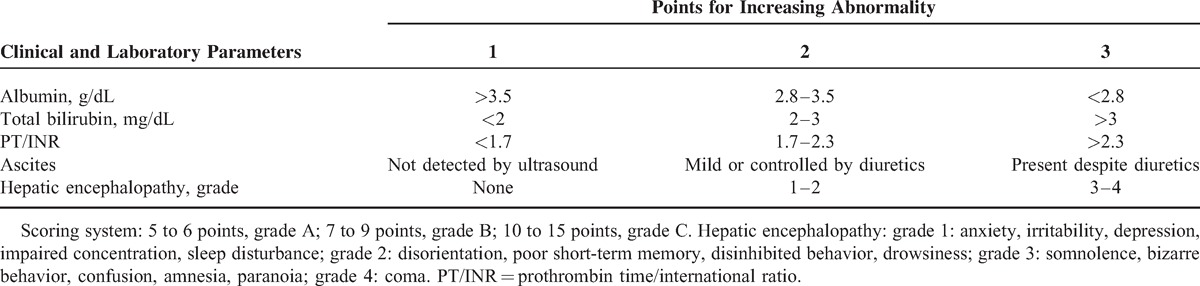
Modified Child–Pugh Score

The number of patients undergoing curative treatments of resection surgery, radiofrequency ablation therapy (RFA), and liver transplantation were 468, 188, and 6 in the A5 group and 85, 104, and 3 in the A6 group, respectively. The number of patients undergoing noncurative treatments of transarterial chemoembolization (TACE), best supportive treatment, chemotherapy, sorafenib, radiotherapy, and chemoradiotherapy were 245, 54, 3, 29, 12, and 11 in the A5 group and 164, 62, 4, 34, 10, and 4 in the A6 group, respectively. The study complied with the standards of the Declaration of Helsinki and current ethical guidelines. The study was also approved by the Institutional Review Board of Taipei Veterans General Hospital.

### Biochemical and Serologic Markers

Serum hepatitis B surface antigen and HCV antibody were tested by radioimmunoassay (Abbott Laboratories, North Chicago, IL) and second-generation enzyme immunoassay (Abbott Laboratories, North Chicago, IL), respectively. Serum biochemistries, including albumin, bilirubin, alanine aminotransferase (ALT), aspartate aminotransferase (AST), alkaline phosphatase (Alk-P), creatinine, glucose, and prothrombin time/international ratio (PT/INR) were measured using a Roche/Hitachi Modular Analytics System (Roche Diagnostics GmbH, Mannheim, Germany). The serum α-fetoprotein (AFP) level was tested using a radioimmunoassay kit (Serono Diagnostic SA, Coinsin, Switzerland).

### Statistical Analysis

The baseline characteristics to be evaluated with the outcomes were selected according to the European Association for the Study of the Liver guidelines published in 2001.^24^ Fisher exact test or χ^2^ test with Yates correction was used to compare categorical variables when appropriate, and the Mann–Whitney *U* test was used to compare continuous variables. Cumulative overall survival rates were estimated by the Kaplan–Meier method and compared using Cox proportional hazards model.

Variables with statistical significance (*P* < 0.05) or proximate to it (*P* < 0.1) by univariate analysis were subjected to multivariate analysis using a forward stepwise logistic regression model. A 2-tailed *P* < 0.05 was considered statistically significant. All statistical analyses were performed using SPSS 17.0 for Windows (SPSS Inc, Chicago, IL).

## RESULTS

### Baseline Clinical Characteristics

The baseline demographic data are shown in Table 2. Patients with HCC in the A6 group were significantly older than those in the A5 group (*P* *=* 0.001). In both groups, men were predominant, but the male-to-female ratio was higher in the A5 group. Patients with HBV carriers were more prevalent in the A5 group than in the A6 group (58.0% vs 43.8%, *P* *=* 0.009), whereas chronic HCV infection was more common in the A6 group (24.1% vs 36.8%, *P* = 0.001). More patients received antiviral therapy in the A5 group than in the A6 group (31.5% vs 24.7%, *P* *=* 0.006).

**TABLE 2:**
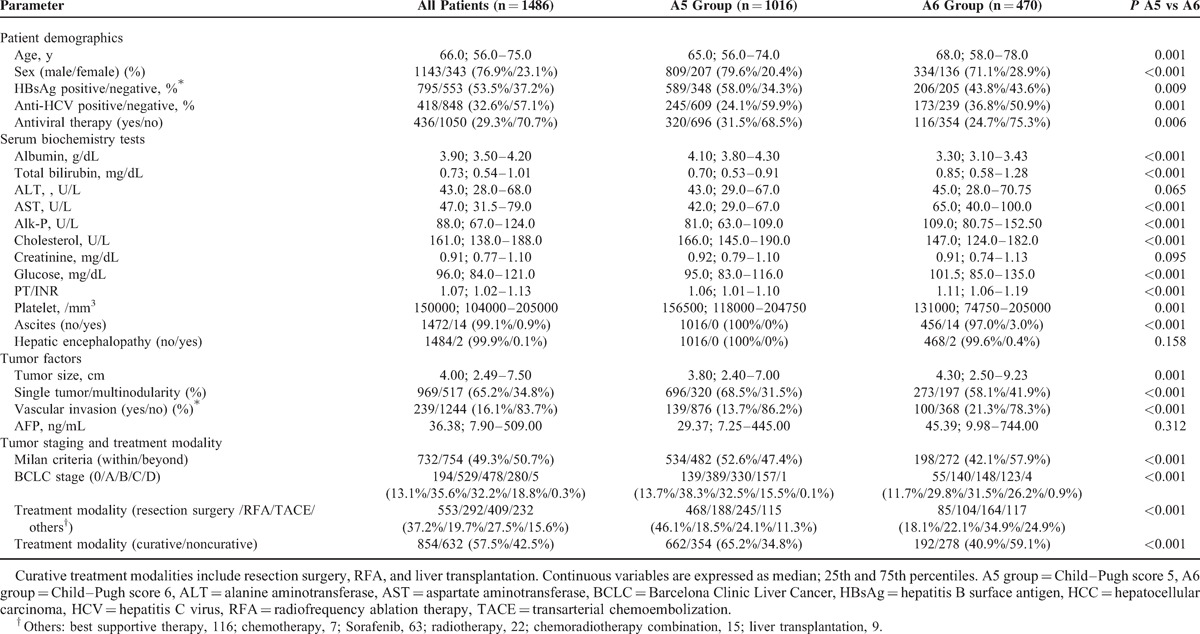
Demographic Data of Child–Pugh Grade A HCC Patients

Liver functional reserve, including albumin, total bilirubin, and PT/INR, was relatively poor for patients in the A6 group (*P* < 0.001). Patients in the A6 group also had lower platelet counts and serum cholesterol levels, and higher serum AST, Alk-P, and glucose levels. In addition, the prevalence rates of ascites and hepatic encephalopathy were both low in the A6 group, at 3.0% and 0.4%, respectively.

Regarding tumor factors, tumor sizes were larger in the A6 group than A5 group (median 4.3 vs 3.8 cm, *P* *=* 0.001), as were the rates of multinodularity (41.9% vs 31.5%, *P* < 0.001) and vascular invasion (21.3% vs 13.7%, *P* < 0.001). The serum AFP levels are comparable in both groups (*P* = 0.312). Compared with the A6 group, more patients in the A5 group satisfied the Milan criteria (52.6% vs 42.1%, *P* < 0.001) and had an earlier BCLC stage (*P* < 0.001). The rate of patients who underwent curative treatment was higher in the A5 group (65.2% vs 40.9%, *P* < 0.001).

### Comparison of Overall Survival Between Patients in the A5 and A6 Groups

**FIGURE 2:**
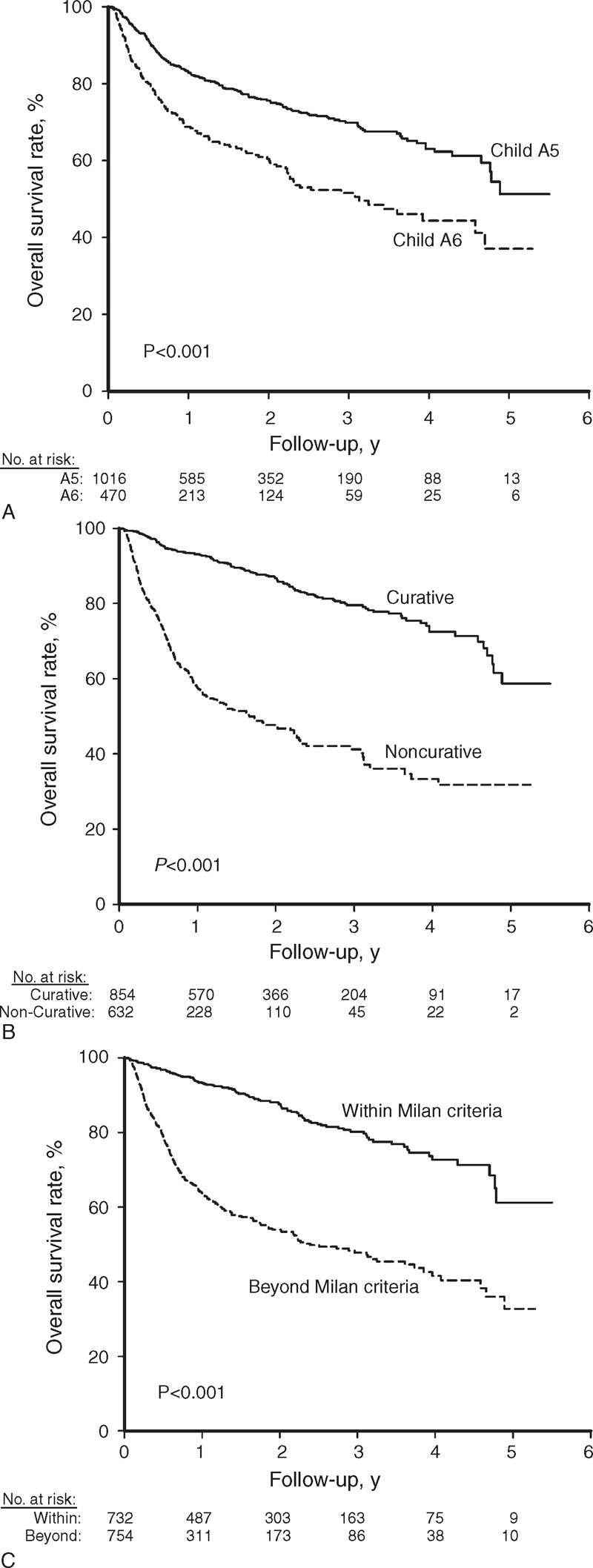
The cumulative curves of overall survival rates stratified by Child–Pugh numeric score, tumor stage, and treatment modality. The overall survival rate was higher in patients who were in the A5 group (A, *P* < 0.001), underwent curative treatment (B, *P* < 0.001), and had tumor stages within the Milan criteria (C, *P* < 0.001). A5 group = Child–Pugh score 5, A6 group = Child–Pugh score 6.

After a median follow-up of 18.6 ± 16.2 months, 397 patients died, leaving 1089 patients still alive on their last visit. As shown in Figure 2A, patients in the A5 group had a significantly higher overall survival rate than those in the A6 group. The cumulative overall survival rates at 1, 2, 3, and 5 years were 83.1%, 75.6%, 69.9%, and 51.3% in the A5 group and 68.8%, 60.5%, 51.5%, and 37.1% in the A6 group, respectively (*P* < 0.001).

**FIGURE 3:**
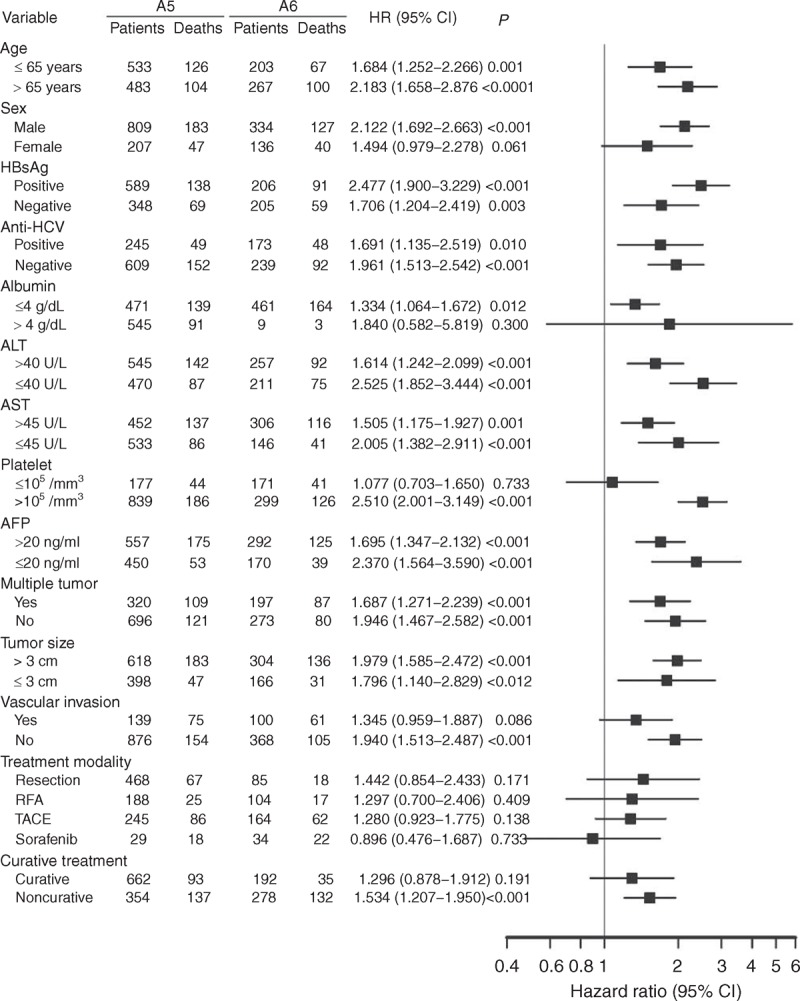
The comparison of overall survival between A5 and A6 groups in stratified analysis by forest plot. A5 group = Child–Pugh score 5, A6 group = Child–Pugh score 6, AFP = α-fetoprotein, ALT = alanine aminotransferase, AST = aspartate aminotransferase, CI = confidence interval, HBsAg = hepatitis B surface antigen, HCV = hepatitis C virus, HR = hazards ratio, RFA = radiofrequency ablation therapy, TACE = transarterial chemoembolization.

**FIGURE 4:**
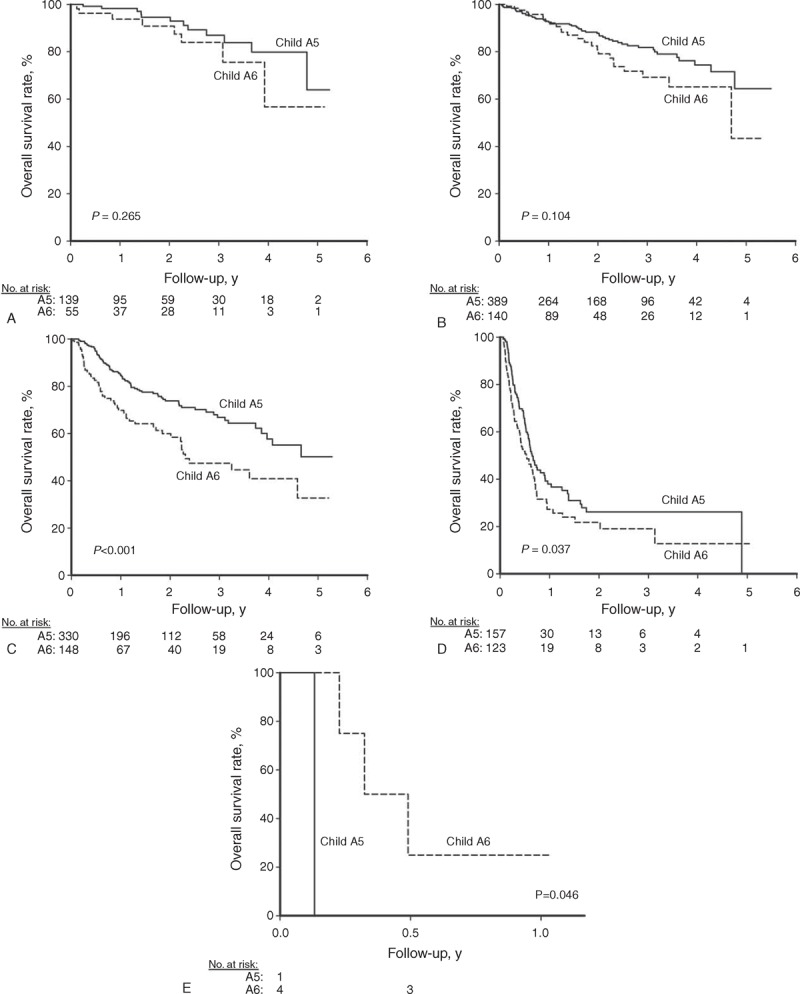
Comparison of cumulative overall survival rates between A5 and A6 groups stratified by the BCLC stage. The overall survival rates were comparable between the A5 and A6 groups in the setting of BCLC stage 0 (A) and stage A (B). However, the overall survival rates were higher in the A5 group compared with the A6 group in patients with BCLC stage B (C), stage C (D), and stage D (E). A5 group = Child–Pugh score 5, A6 group = Child–Pugh score 6, BCLC = Barcelona Clinic Liver Cancer.

We further compared the prognosis between these 2 groups using subgroup analysis. As shown in Figure 3, the overall survival rates were higher in the A5 group in most subgroups, except for female patients, those with serum albumin > 4 g/dL, platelet ≤10^5^/mm^3^, or the presence of vascular invasion, and in patients who underwent curative treatment modalities. When stratified by BCLC stage, patients in the A5 group had similar prognoses compared with those in the A6 group in the setting of BCLC stages 0 and A (Figure 4A and B). In patients with advanced tumor stages (BCLC stages B–D), the overall survival rates were higher in the A5 group (Figure 4C–E).

### Multivariate Analysis of Independent Risk Factors Associated With Poor Prognosis

As shown in Table 3, multivariate analysis showed that the independent risk factors associated with poor overall survival in Child–Pugh grade A patients with HCC were non-HCV carrier (hazard ratio [HR] 1.590, *P* < 0.001), albumin levels ≤4 g/dL (HR 1.610, *P* < 0.001), AST >45 U/L (HR 1.667, *P* < 0.001), AFP >20 ng/mL (HR 2.046, *P* < 0.001), multinodularity (HR 1.288, *P* = 0.033), tumor size > 3 cm (HR 1.797, *P* < 0.001), the presence of vascular invasion (HR 2.646, *P* < 0.001), and noncurative treatment (HR 2.498, *P* < 0.001). Notably, the overall survival rate was significantly higher in patients in the A5 group compared with those in the A6 group in most subgroup analyses by univariate analysis. Child–Pugh numeric score was not an independent risk factor of prognoses in HCC patients with Child–Pugh grade A after adjusting for confounding factors by multivariate analysis.

**TABLE 3:**
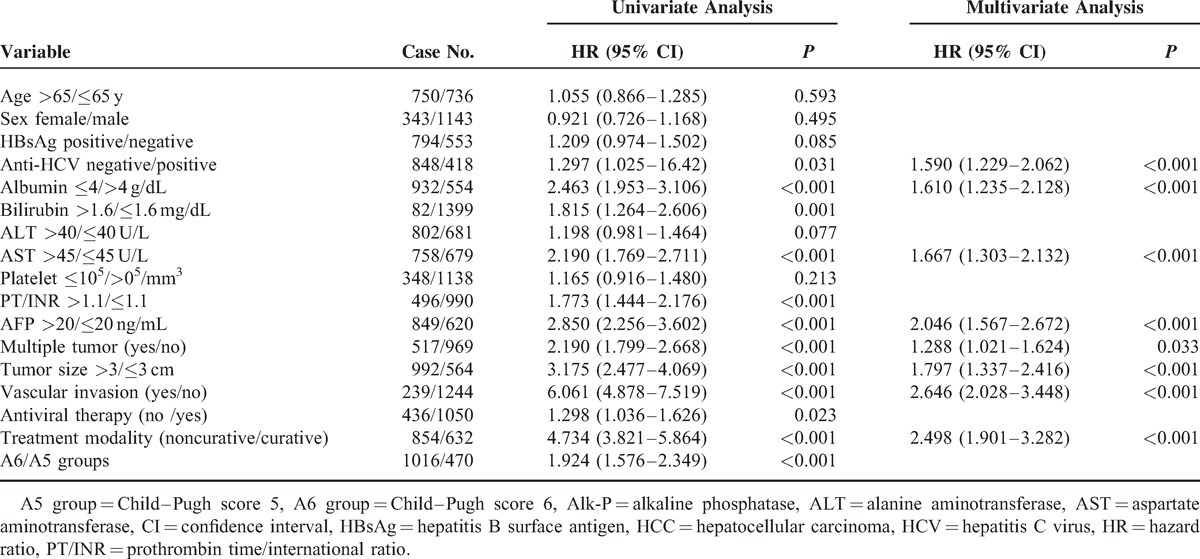
Factors Associated With Poor Overall Survival in Child–Pugh Grade A and HCC

### Comparison of Prognosis Between A5 and A6 Groups Stratified by Tumor Stage and Treatment Modality

A total of 854 (57.5%) patients underwent curative therapies, and the remaining 632 (42.5%) patients received noncurative therapy. As shown in Figure 2B, those who underwent curative therapy had a significantly higher overall survival rate than their counterparts. The cumulative overall survival rates at 1, 2, 3, and 5 years were 93.2%, 86.7%, 79.5%, and 58.7% in the curative group and 57.7%, 47.7%, 41.2%, and 31.8% in the noncurative group, respectively (*P* < 0.001).

When stratified by the tumor stage, 732 (49.3%) and 754 (50.7%) patients had HCC within and beyond the Milan criteria, respectively. The cumulative overall survival rates at 1, 2, 3, and 5 years were 93.4%, 87.5%, 80.2%, and 61.2% for patients within the Milan criteria and 63.7%, 53.9%, 47.8%, and 32.7% in patients beyond the Milan criteria, respectively (Figure 2C, *P* < 0.001).

**FIGURE 5:**
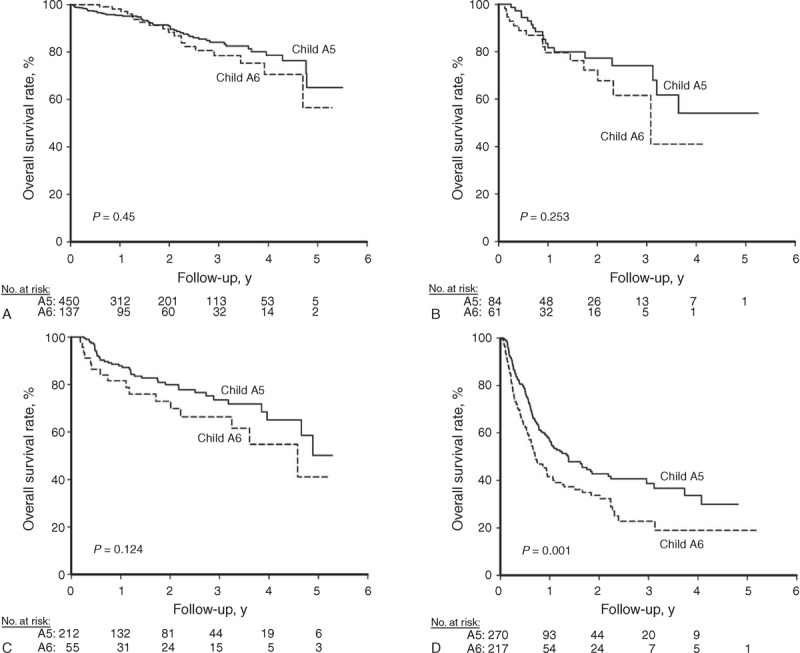
The comparison of cumulative overall survival rates between A5 and A6 groups stratified by tumor stage and treatment modality. (A) Patients with tumors within the Milan criteria who underwent curative therapy, (B) patients with tumors within the Milan criteria who underwent noncurative therapy, (C) patients with tumors beyond the Milan criteria who underwent curative therapy, and (D) patients with tumors beyond the Milan criteria who underwent noncurative therapy. A5 group = Child–Pugh score 5, A6 group = Child–Pugh score 6.

In patients within the Milan criteria, the overall survival rates were comparable between the A5 and A6 groups, irrespective of the treatment modalities (Figure 5A and B). For patients who underwent curative therapies, the cumulative overall survival rates at 3 and 5 years were 84.2% and 65.0% in the A5 group and 78.5% and 56.5% in the A6 group, respectively (*P* = 0.450). The cumulative overall survival rates of the noncurative treatment group at 3 and 5 years were 74.1% and 54.0% in the A5 group and 61.6% and 41.1% in the A6 group, respectively (*P* = 0.253).

Regarding patients who had tumors beyond the Milan criteria, the overall survival showed no significant statistical difference between the A5 and A6 groups in the setting of curative therapies (Figure 5C, *P* = 0.124). The cumulative overall survival rates at 3 and 5 years were 73.5% and 50.2% in the A5 group and 66.4% and 41.1% in the A6 group, respectively. However, the overall survival rate showed a significant statistical difference between the A5 and A6 patients in the noncurative group (Figure 5D, *P* = 0.001). The cumulative overall survival rates at 3 and 5 years were 38.8% and 29.9% in the A5 group and 22.7% and 19.0% in the A6 group, respectively.

## DISCUSSION

In this study, overall survival was significantly different with only 1 score difference (A5 vs A6) in HCC patients with Child–Pugh grade A according to univariate analysis and in most subgroup analyses. However, the Child–Pugh numeric score was not an independent risk factor of prognosis according to multivariate analysis. Moreover, when we stratified these patients by tumor stage and treatment modality, the overall survival rates were similar between these 2 groups of patients, except for those who had tumors beyond the Milan criteria and who had undergone noncurative treatment. This result supports the hypothesis that an intensive and curative treatment may provide a better long-term survival rate in HCC patients with Child–Pugh grade A.^25^

In our cohort, there were significant discrepancies in terms of demographic characteristics, tumor factors, liver functional reserve, and viral etiologies between these 2 groups. Compared with those in the A6 group, patients in the A5 group were younger and predominantly men, and they had more HBV infection, less HCV carrier, less vascular invasion, higher proportions of tumor stages within the Milan criteria, and higher rates of undergoing curative therapies. In the present study, lower serum albumin levels, higher AST levels, higher serum AFP levels, multinodularity, larger tumor size, the presence of vascular invasion, and noncurative treatment modalities were associated with poorer overall survival in HCC treatment. These factors have been confirmed by previous studies.^26–31^ However, A5 versus A6 groups were not shown to be an independent risk factor regarding overall survival after adjusting for confounding prognostic factors by multivariate analysis. The poor overall survival in the A6 group may be attributed to advanced tumor factors and the selection of treatment modality but not poor liver functional reserve because liver function was relatively well preserved in patients with Child–Pugh grade A.

Whether viral etiology determines the outcomes in HCC patients is still controversial.^32–36^ In our current study, patients with chronic HCV infection had a better overall survival rate than their counterparts. We conducted further analyses and found that patients with HCV infection were associated with older age, relatively active hepatic necroinflammation, and poorer liver function reserve (Table 4). In contrast, regarding the tumor factors, HCV carriers had smaller tumor size, less vascular invasion, and earlier tumor stage. Therefore, we propose that due to the effect of liver functional reserve being restricted in Child–Pugh stage A patients in the current study, tumor factors seemed to affect the overall survival rate more prominently, which in turn led to better prognoses in patients with chronic hepatitis C.

**TABLE 4:**
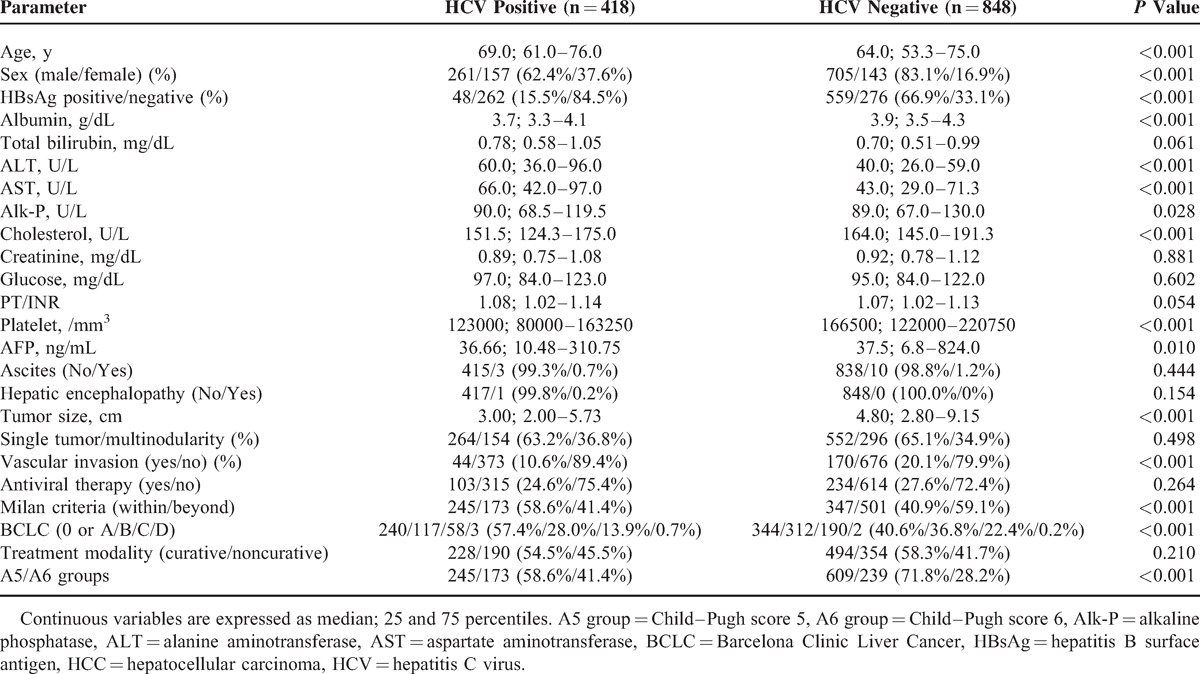
Comparison of Demographic Data Between HCV Carrier and Non-HCV Carrier in Child–Pugh Grade A HCC Patients

Surgical resection, RFA, and liver transplantation are recommended as the first-line curative treatment modalities in early stage BCLC.^16^ Among the curative treatments, resection surgery and RFA are the major therapies in daily practice in Taiwan because of organ shortage.^37,38^ According to the BCLC guidelines, curative treatment is not suggested for patients with HCC beyond the Milan criteria due to high tumor recurrence, greater surgical complications, and lower long-term outcomes,^16^ However, previous studies have demonstrated that resection surgery provides better prognoses than TACE for HCC patients beyond the Milan criteria.^39–41^ Moreover, resection is reported to be as safe as TACE for such patients.^39^ Therefore, resection surgery may be considered as the priority treatment for patients with a tumor stage beyond the Milan criteria if there are no contraindications for surgery.

In case of tumors beyond the Milan criteria in the present study, the overall survival rates were similar between the A5 and A6 groups when they underwent curative treatment. However, when patients underwent noncurative treatment, the survival rate was significantly poorer in the A6 group. This result suggests that intensive and curative treatment may be indicated in HCC patients with A6 group, even if the tumor is beyond the Milan criteria.

Treatment modalities are highly associated with long-term survival and proposed to be an independent risk factor for HCC patients.^42^ However, the selection of treatment may be affected by many confounding factors, including tumor factors, performance status, and availability of treatment modalities.^43^ In the present study, 80.9% of the HCC patients were Child–Pugh grade A. However, only around 57.5% of the patients with Child–Pugh grade A received curative treatment. The proportion of Child–Pugh grade A and the curative treatment rate were similar to that in other groups.^44^ In 1 large cohort study from Korea, Kim et al^44^ enrolled 1717 HCC patients and showed that more than half of the patients with early stage HCC underwent TACE, rather than resection or local ablation therapy. Another meta-analysis conducted on patients from the United States also demonstrated that the pooled rate of curative treatment was only 59.0% in HCC patients who were diagnosed at an early stage.^6^ Although the BCLC staging system and its recommended treatments are suggested by the current guidelines for HCC, the treatment modality does not match exactly with the guidelines in the real world, and underutilization of treatment is common in daily practice.^44,45^ This might affect the long-term outcomes of HCC patients.^43^ To improve their prognoses, it may be warranted to encourage patients to undergo curative therapies if their liver functions are well preserved. A multidisciplinary approach may also help clinical physicians and patients to choose the optimal treatment modalities.^6^

With the increasing incidences of obesity globally, metabolic disorders and NASH have now been important etiologies of HCC.^46^ Welzel et al^46^ further demonstrated that although HBV or HCV infection had a higher odds ratio as a risk factor for HCC, diabetes and/or obesity had the largest population-attributable fraction for HCC with a value of 36.6%, suggesting a dominant role for diabetes and obesity in hepatic carcinogenesis. In our study, although chronic HBV or HCV infections were the major etiologies of HCC, NASH was the major cause among the nonviral etiologies (184/321, 57.3%). Of note, growing evidence shows that HCC could develop in patients with NASH in the absence of apparent cirrhosis, especially among men.^47,48^ It is crucial to search for and identify the risk factors predicting disease progression and HCC in patients with NASH irrespective of cirrhotic status. Moreover, adequate surveillance for HCC might be needed for the high-risk group of patients.

There are few limitations regarding this study that need to be addressed. First, the study included HCC patients from a single tertiary center. Twenty-five percent of the patients were excluded because they did not have sufficiently complete data for the Child–Pugh score calculation. Moreover, previous studies suggested that blood ammonia levels could be served as a reliable surrogate marker of hepatic decompensation, portal hypertension, and liver functional reserve.^23^ However, we did not have the data of ammonia levels. Further studies are warranted to elucidate the role of noninvasive serum markers on the prognoses of patients with HCC. Second, interobserver bias may exist in the amount of ascites and the degree of hepatic encephalopathy for Child–Pugh score calculation. Minimal ascites and mild hepatic encephalopathy may be missing. Lastly, liver transplantation was performed in only 0.6% of the patients in our cohort because of a local organ shortage. The data maybe not be applied to centers with a high-volume of liver transplantation.

## CONCLUSIONS

HCC patients with A5 group had better prognoses than those with A6 group. However, tumor factors and treatment modalities were more important than Child–Pugh numeric scores when examining Child–Pugh grade A patients.

## References

[1] Ferenci P Fried M Labrecque D Hepatocellular carcinoma (HCC): a global perspective. *J Clin Gastroenterol* 2010; 44:239–245. 20216082 10.1097/MCG.0b013e3181d46ef2

[2] El-Serag HB Epidemiology of viral hepatitis and hepatocellular carcinoma. *Gastroenterology* 2012; 142:1264–1273. 22537432 10.1053/j.gastro.2011.12.061 PMC3338949

[3] Kee KM Wang JH Lin CY Validation of the 7th edition TNM staging system for hepatocellular carcinoma: an analysis of 8,828 patients in a single medical center. *Dig Dis Sci* 2013; 58:2721–2728. 23703450 10.1007/s10620-013-2716-8

[4] Fan ST Mau Lo C Poon RT Continuous improvement of survival outcomes of resection of hepatocellular carcinoma: a 20-year experience. *Ann Surg* 2011; 253:745–758. 21475015 10.1097/SLA.0b013e3182111195

[5] Han KH Kim do Y Park JY Survival of hepatocellular carcinoma patients may be improved in surveillance interval not more than 6 months compared with more than 6 months: a 15-year prospective study. *J Clin Gastroenterol* 2013; 47:538–544. 23340065 10.1097/MCG.0b013e3182755c13

[6] Tan D Yopp A Beg MS Meta-analysis: underutilisation and disparities of treatment among patients with hepatocellular carcinoma in the United States. *Aliment Pharmacol Ther* 2013; 38:703–712. 23957569 10.1111/apt.12450 PMC3777750

[7] Kwak HW Park JW Nam BH Clinical outcomes of a cohort series of patients with hepatocellular carcinoma in a hepatitis B virus-endemic area. *J Gastroenterol Hepatol* 2014; 29:820–829. 24325272 10.1111/jgh.12470

[8] Kao WY Chiou YY Hung HH Younger hepatocellular carcinoma patients have better prognosis after percutaneous radiofrequency ablation therapy. *J Clin Gastroenterol* 2012; 46:62–70. 21934530 10.1097/MCG.0b013e31822b36cc

[9] Su CW Chiou YW Tsai YH The influence of hepatitis B viral load and pre-S deletion mutations on post-operative recurrence of hepatocellular carcinoma and the tertiary preventive effects by anti-viral therapy. *PloS One* 2013; 8:e66457. 23805222 10.1371/journal.pone.0066457 PMC3689837

[10] Nault JC De Reynies A Villanueva A A hepatocellular carcinoma 5-gene score associated with survival of patients after liver resection. *Gastroenterology* 2013; 145:176–187. 23567350 10.1053/j.gastro.2013.03.051

[11] Su CW Lei HJ Chau GY The effect of age on the long-term prognosis of patients with hepatocellular carcinoma after resection surgery: a propensity score matching analysis. *Arch Surg* 2012; 147:137–144. 22006855 10.1001/archsurg.2011.288

[12] Mehta N Fidelman N Sarkar M Factors associated with outcomes and response to therapy in patients with infiltrative hepatocellular carcinoma. *Clin Gastroenterol Hepatol* 2013; 11:572–578. 23333661 10.1016/j.cgh.2012.12.030 PMC4052891

[13] Lee YH Hsu CY Huang YH Vascular invasion in hepatocellular carcinoma: prevalence, determinants and prognostic impact. *J Clin Gastroenterol* 2014; 48:734–741. 24100755 10.1097/MCG.0b013e3182a8a254

[14] Marrero JA Fontana RJ Barrat A Prognosis of hepatocellular carcinoma: comparison of 7 staging systems in an American cohort. *Hepatology* 2005; 41:707–716. 15795889 10.1002/hep.20636

[15] Yau T Tang VY Yao TJ Development of Hong Kong Liver Cancer staging system with treatment stratification for patients with hepatocellular carcinoma. *Gastroenterology* 2014; 146:1691–1700. 24583061 10.1053/j.gastro.2014.02.032

[16] de Lope CR Tremosini S Forner A Management of HCC. *J Hepatol* 2012; 56 suppl 1:S75–87. 22300468 10.1016/S0168-8278(12)60009-9

[17] Kinoshita A Onoda H Imai N The Glasgow Prognostic Score, an inflammation based prognostic score, predicts survival in patients with hepatocellular carcinoma. *BMC Cancer* 2013; 13:52. 23374755 10.1186/1471-2407-13-52 PMC3571892

[18] Piscaglia F Terzi E Cucchetti A Treatment of hepatocellular carcinoma in Child–Pugh B patients. *Dig Liver Dis* 2013; 45:852–858. 23582346 10.1016/j.dld.2013.03.002

[19] Kudo M Osaki Y Matsunaga T Hepatocellular carcinoma in Child–Pugh C cirrhosis: prognostic factors and survival benefit of nontransplant treatments. *Dig Dis* 2013; 31:490–498. 24281026 10.1159/000355259

[20] Kim HY Park JW Joo J Worse outcome of sorafenib therapy associated with ascites and Child–Pugh score in advanced hepatocellular carcinoma. *J Gastroenterol Hepatol* 2013; 28:1756–1761. 23800278 10.1111/jgh.12310

[21] Nouso K Ito Y Kuwaki K Prognostic factors and treatment effects for hepatocellular carcinoma in Child C cirrhosis. *Br J Cancer* 2008; 98:1161–1165. 18349849 10.1038/sj.bjc.6604282 PMC2359634

[22] Bruix J Sherman M Management of hepatocellular carcinoma. *Hepatology* 2005; 42:1208–1236. 16250051 10.1002/hep.20933

[23] Tarantino G Citro V Esposito P Blood ammonia levels in liver cirrhosis: a clue for the presence of portosystemic collateral veins. *BMC Gastroenterol* 2009; 9:21. 19292923 10.1186/1471-230X-9-21 PMC2662872

[24] Bruix J Sherman M Llovet JM Clinical management of hepatocellular carcinoma. Conclusions of the Barcelona-2000 EASL conference. European Association for the Study of the Liver. *J Hepatol* 2001; 35:421–430. 11592607 10.1016/s0168-8278(01)00130-1

[25] Giannini EG Savarino V Farinati F Influence of clinically significant portal hypertension on survival after hepatic resection for hepatocellular carcinoma in cirrhotic patients. *Liver Int* 2013; 33:1594–1600. 23654354 10.1111/liv.12199

[26] Tandon P Garcia-Tsao G Prognostic indicators in hepatocellular carcinoma: a systematic review of 72 studies. *Liver Int* 2009; 29:502–510. 19141028 10.1111/j.1478-3231.2008.01957.x PMC2711257

[27] N’Kontchou G Mahamoudi A Aout M Radiofrequency ablation of hepatocellular carcinoma: long-term results and prognostic factors in 235 Western patients with cirrhosis. *Hepatology* 2009; 50:1475–1483. 19731239 10.1002/hep.23181

[28] Waki K Aikata H Katamura Y Percutaneous radiofrequency ablation as first-line treatment for small hepatocellular carcinoma: results and prognostic factors on long-term follow up. *J Gastroenterol Hepatol* 2010; 25:597–604. 20074153 10.1111/j.1440-1746.2009.06125.x

[29] Yang T Zhang J Lu JH A new staging system for resectable hepatocellular carcinoma: comparison with six existing staging systems in a large Chinese cohort. *J Cancer Res Clin Oncol* 2011; 137:739–750. 20607551 10.1007/s00432-010-0935-3 PMC11827787

[30] Lim KC Chow PK Allen JC Microvascular invasion is a better predictor of tumor recurrence and overall survival following surgical resection for hepatocellular carcinoma compared to the Milan criteria. *Ann Surg* 2011; 254:108–113. 21527845 10.1097/SLA.0b013e31821ad884

[31] Ishizawa T Hasegawa K Aoki T Neither multiple tumors nor portal hypertension are surgical contraindications for hepatocellular carcinoma. *Gastroenterology* 2008; 134:1908–1916. 18549877 10.1053/j.gastro.2008.02.091

[32] Chen PH Kao WY Chiou YY Comparison of prognosis by viral etiology in patients with hepatocellular carcinoma after radiofrequency ablation. *Ann Hepatol* 2013; 12:263–273. 23396738

[33] Kao WY Su CW Chau GY A comparison of prognosis between patients with hepatitis B and C virus-related hepatocellular carcinoma undergoing resection surgery. *World J Surg* 2011; 35:858–867. 21207029 10.1007/s00268-010-0928-z

[34] Franssen B Alshebeeb K Tabrizian P Differences in surgical outcomes between hepatitis B- and hepatitis C-related hepatocellular carcinoma: a retrospective analysis of a single North American center. *Ann Surg* 2014; 260:650–658. 25203882 10.1097/SLA.0000000000000917

[35] Lee JJ Kim PT Fischer S Impact of viral hepatitis on outcomes after liver resection for hepatocellular carcinoma: results from a North American center. *Ann Surg Oncol* 2014; 21:2708–2716. 24806113 10.1245/s10434-014-3609-6

[36] Cantarini MC Trevisani F Morselli-Labate AM Effect of the etiology of viral cirrhosis on the survival of patients with hepatocellular carcinoma. *Am J Gastroenterol* 2006; 101:91–98. 16405539 10.1111/j.1572-0241.2006.00364.x

[37] Hung HH Chiou YY Hsia CY Survival rates are comparable after radiofrequency ablation or surgery in patients with small hepatocellular carcinomas. *Clin Gastroenterol Hepatol* 2011; 9:79–86. 20831902 10.1016/j.cgh.2010.08.018

[38] Wu WC Chiou YY Hung HH Prognostic significance of computed tomography scan-derived splenic volume in hepatocellular carcinoma treated with radiofrequency ablation. *J Clin Gastroenterol* 2012; 46:789–795. 22941428 10.1097/MCG.0b013e31825ceeb5

[39] Hsu CY Hsia CY Huang YH Comparison of surgical resection and transarterial chemoembolization for hepatocellular carcinoma beyond the Milan criteria: a propensity score analysis. *Ann Surg Oncol* 2012; 19:842–849. 21913008 10.1245/s10434-011-2060-1

[40] Yamashita Y Taketomi A Shirabe K Outcomes of hepatic resection for huge hepatocellular carcinoma (≥10 cm in diameter). *J Surg Oncol* 2011; 104:292–298. 21465490 10.1002/jso.21931

[41] Liu PH Lee YH Hsia CY Surgical resection versus transarterial chemoembolization for hepatocellular carcinoma with portal vein tumor thrombosis: a propensity score analysis. *Ann Surg Oncol* 2014; 21:1825–1833. 24499831 10.1245/s10434-014-3510-3

[42] Hsu CY Lee YH Hsia CY Performance status in patients with hepatocellular carcinoma: determinants, prognostic impact, and ability to improve the Barcelona Clinic Liver Cancer system. *Hepatology* 2013; 57:112–119. 22806819 10.1002/hep.25950

[43] Kanwal F Befeler A Chari RS Potentially curative treatment in patients with hepatocellular cancer—results from the liver cancer research network. *Aliment Pharmacol Ther* 2012; 36:257–265. 22670798 10.1111/j.1365-2036.2012.05174.x

[44] Kim BK Kim SU Park JY Applicability of BCLC stage for prognostic stratification in comparison with other staging systems: single centre experience from long-term clinical outcomes of 1717 treatment-naive patients with hepatocellular carcinoma. *Liver Int* 2012; 32:1120–1127. 22524688 10.1111/j.1478-3231.2012.02811.x

[45] Bolondi L Burroughs A Dufour JF Heterogeneity of patients with intermediate (BCLC B) hepatocellular carcinoma: proposal for a subclassification to facilitate treatment decisions. *Semin Liver Dis* 2012; 32:348–359. 23397536 10.1055/s-0032-1329906

[46] Welzel TM Graubard BI Quraishi S Population-attributable fractions of risk factors for hepatocellular carcinoma in the United States. *Am J Gastroenterol* 2013; 108:1314–1321. 23752878 10.1038/ajg.2013.160 PMC4105976

[47] Baffy G Brunt EM Caldwell SH Hepatocellular carcinoma in non-alcoholic fatty liver disease: an emerging menace. *J Hepatol* 2012; 56:1384–1391. 22326465 10.1016/j.jhep.2011.10.027

[48] Yasui K Hashimoto E Komorizono Y Characteristics of patients with nonalcoholic steatohepatitis who develop hepatocellular carcinoma. *Clin Gastroenterol Hepatol* 2011; 9:428–433. 21320639 10.1016/j.cgh.2011.01.023

